# Obtaining Gene-Modified HLA-E-Expressing Feeder Cells for Stimulation of Natural Killer Cells

**DOI:** 10.3390/pharmaceutics16010133

**Published:** 2024-01-19

**Authors:** Nadezhda A. Alekseeva, Maria A. Streltsova, Julia D. Vavilova, Maria O. Ustiuzhanina, Anastasia I. Palamarchuk, Anna A. Boyko, Nikita D. Timofeev, Alexey I. Popodko, Elena I. Kovalenko

**Affiliations:** 1 Shemyakin and Ovchinnikov Institute of Bioorganic Chemistry, Russian Academy of Sciences, ul. Miklukho-Maklaya, 16/10, 117997 Moscow, Russia; nadalex@inbox.ru (N.A.A.); mstreltsova@mail.ru (M.A.S.); juliateterina12@gmail.com (J.D.V.); mashaust1397@gmail.com (M.O.U.); palanastasia@yandex.ru (A.I.P.); boyko_anna@mail.ru (A.A.B.); ndtimofeev@gmail.com (N.D.T.); 2 Department of Radiation Oncology, European Medical Center, Schepkina 35, 129110 Moscow, Russia; popodko.alexey@gmail.com

**Keywords:** NK cells, HLA-E, feeder cells, CD57-negative, NK cell expansion

## Abstract

Human cytomegalovirus (HCMV)-specific adaptive NK cells are capable of recognizing viral peptides presented by HLA-E on infected cells via the NKG2C receptor. Using retroviral transduction, we have generated a K562-cell-based line expressing HLA-E in the presence of the HLA-E-stabilizing peptide, which has previously shown the capacity to enhance adaptive NK cell response. The obtained K562-21E cell line was employed to investigate proliferative responses of the CD57^−^ NK cell subset of HCMV-seropositive and seronegative donors. Stimulation of CD57^−^ NK cells with K562-21E/peptide resulted in an increased cell expansion during the 12-day culturing period, regardless of the serological HCMV status of the donor. The enhanced proliferation in response to the peptide was associated with a greater proportion of CD56^bright^HLA-DR^+^ NK cells. In later stages of cultivation, the greatest proliferative response to K562-21E/peptide was shown for a highly HCMV-seropositive donor. These expanded NK cells were characterized by the accumulation of CD57^−^KIR2DL2/3^+^NKG2C^+^NKG2A^−^ cells, which are hypothesized to represent adaptive NK cell progenitors. The K562-21E feeder cells can be applied both for the accumulation of NK cells as therapeutic effectors, and for the study of NK cell maturation into the adaptive state after the HLA-E peptide presentation.

## 1. Introduction

Natural killer (NK) cells are cytotoxic lymphocytes of the innate immune system. NK cells have intrinsic antitumor capabilities, which are controlled by a wide range of activating/costimulatory and inhibitory receptors. Due to these properties, NK cells represent an attractive agent for cancer cell therapy [1]. Although NK cells are capable of recognizing and killing without prior sensitization, their functional activity can be significantly increased during activation, which is essential for NK-cell-based adoptive immunotherapy [2]. In addition, given the low content of NK cells in peripheral blood (5–20% of lymphocytes) and their short lifespan after infusion, in vitro expansion is needed to obtain large numbers of functionally active NK cells for effective adoptive immunotherapy [3]. The primary stimuli for NK cell activation are cytokines, such as IL-2, IL-12, IL-18, IL-15, and IL-21 [4,5]. However, it has recently been shown that the highest expansion rates of NK cells can be achieved with the simultaneous stimulation of NK cells with cytokines and feeder cells, which can be genetically modified [6,7,8,9]. The K562 cell line, deficient in HLA class I molecules, provides an excellent basis for feeder cells. K562 cells are capable of inducing an intense cytolytic NK cell response; therefore, they are used as standard target cells in a variety of cytotoxic assays [3]. Several genetically modified K562 cell line variants are known, particularly those expressing the membrane-bound cytokines [10,11,12]. K562-based cells expressing mbIL-21, in combination with IL-2, have been shown to be among the most effective feeder cells for inducing NK cell expansion. Notably, additional feeder cell modification with mbIL-15 did not result in a substantial increase in the expansion rate or in changes in the phenotype (KIR, CD16, and NKG2D) and cytotoxicity of NK cells [12]. While IL-15 is known to act more as a survival factor and is not strictly required for memory-like NK cell differentiation [13], IL-21 plays a role in the proliferation and maturation of NK cells and, in combination with IL-2, enhances the expression of intracellular effector molecules, such as granzyme B and perforin, both at the mRNA and protein levels [14,15]. However, expansion rates may vary in different NK cell subsets. In particular, CD57^+^ NK cells contain low numbers of cells capable of proliferating in response to K562-mbIL21 cells and IL-2 [16]. Moreover, proliferative NK cell responses to feeder cells and interleukins, regardless of their combination, vary among donors. This variation may be associated with the individual heterogeneity of the NK cell population.

The HLA-E molecule is a ligand for many NK cell receptors of the CD94/NKG2 family. In this family, the most widespread receptors are the inhibitory receptor NKG2A and the activating receptor NKG2C. NKG2A is highly expressed on less differentiated NK cells [17], while the accumulation of adaptive-like NKG2C^+^ NK cells often occurs during widespread lifelong human cytomegalovirus (HCMV) infections [18]. The surface expression of HLA-E is stabilized by 9-mer peptides, usually derived from leader peptides of HLA-A, B, C, G proteins containing the consensus sequence of VM(A/P)PRT(L/V)(V/L/I/F)L [19]. Peptides presented by HLA-E can modulate the binding affinity of HLA-E to the receptors. In particular, the peptide VMAPRTLFL has been shown to increase the binding affinity of HLA-E to NKG2C, which enhances the adaptive response of NK cells [20,21]. This peptide is homologous to a region of the HLA-G molecule’s leader sequence (known for its limited distribution in tissues), as well as to a region of the UL40 protein of some HCMV strains [20]. It has been shown that a slight change in the structure of the peptide can lead to a strong impairment in the formation of the HLA-E-NKG2C complex [21]. Adaptive NKG2C^+^NK cells not only may help to control the progression of HCMV infection [22,23,24], but also exhibit significant antitumor potential. In particular, NKG2C^+^ NK cell expansion in patients with leukemia after hematopoietic stem cell transplantation (HSCT) was associated with reduced relapse rates and better clinical outcomes of the disease [25,26]. The effectiveness of adaptive NK cell applications may be related to both the increase in alloreactive KIR-expressing NK cells with reduced levels of NKG2A expression [27], and their ability to mediate the ADCC response [28]. Stimulation of HCMV-specific NKG2C^+^ NK cells may enhance their cytotoxic potential and lifespan. Hence, in order to address the challenge of developing high numbers of highly efficient NK-cell-based effectors, it is crucial to obtain feeder cells capable of presenting HCMV peptides via HLA-E to NK cells, and to study the properties of NK cell cultures after stimulation. Knowledge of the specific phenotypes that distinguish more responsive cells may aid in the selection of donors for NK-cell-based adoptive cell therapy.

In this work, we inserted, using retroviral transduction, the HLA-E gene into the K562 line expressing membrane-bound IL-21, which induced the active proliferation of NK cells to obtain feeder cells expressing HLA-E on their surface (K562-21E cells). In humans, there are only two HLA-E alleles, which differ in one amino acid at position 107 in the protein sequence [29,30]. Allele *0101 (HLA-E^R^) has arginine, allele *0103 (HLA-E^G^) contains glycine in this position [29]. HLA-E^G^ has a higher peptide affinity, higher surface expression, and higher thermostability of the corresponding protein, moreover, it is more ancient than HLA-E^R^ [29]. Allele 0103 (HLA-E^G^) was selected for the assembly of the retroviral construct. The VMAPRTLFL (LFL) peptide, previously shown to increase the affinity of HLA-E binding to the NKG2 receptors [21], was synthesized.

We detected surface HLA-E on K562-21E cells exclusively upon exogenous addition of the LFL peptide. After irradiation of the newly modified feeder cells, we analyzed their ability to activate the less differentiated CD57^−^ NK cell subset. CD57-negative NK cells have a higher proliferative potential than CD57^+^ NK cells [31] and are of significant interest for immunotherapy. NK cells and, in particular, their CD57^−^ subset are characterized by a high heterogeneity. CD57-negative cells contain a pool of possible precursors of adaptive NK cells with a CD56^dim^CD57^−^NKG2C^+^ phenotype [32]. Moreover, previously we have shown that activated and proliferating CD57^−^ NK cells are more susceptible to retroviral transduction [33], which unveils the possibility of obtaining genetically modified NK cells with enhanced antitumor properties. As a result, the response of CD57^−^ NK cells to the HCMV peptide (LFL) presentation may vary depending on the subset. Obtaining HLA-E-expressing feeder cells opens up the possibility of their use in the peptide-mediated response of NK cells at the subset level. We have performed a comparative analysis of the expansion level, phenotype, and functional activity of CD57^−^ NK cells from seven individuals with various levels of NKG2C^+^ cells and different HCMV serological statuses. The analysis was conducted upon pulse stimulation with an HLA-E-expressing K562 cell line in the presence or absence of the synthetic peptide VMAPRTLFL. The analysis of the relationship between the phenotypic characteristics of ex vivo NK cells and their subsequent expansion rates was carried out.

## 2. Materials and Methods

### 2.1. Construction of HLA-E-Containing Vector

Total RNA was isolated from peripheral blood mononuclear cells (PBMC) of a healthy donor. cDNA synthesis was carried out using a standard 18dT primer that is specific for the 3’ end of mRNAs. To amplify the HLA-E^G^ gene, the primers were selected with BamHI and EcoRI restriction sites at their ends: forward—5′-GCAAGGATCCATGGTAGATGGAACCCTC, reverse—5′-GACTGAATTCTTACAAGCTGTGAGACTCAGAC. After amplification, the HLA-E product was purified using Cleanup St PCR (Evrogen, Moscow, Russia). The PCR fragment and plasmid pBABE GFP (Addgene #10668) were treated with endonucleases BamHI and EcoRI (Sibenzyme, Novosibirsk, Russia) for subsequent ligation of the fragments with T4-ligase (Sibenzyme, Novosibirsk, Russia). After transformation, the individual *E. coli* colonies were screened by PCR and positive HLA-E clones were expanded. The HLA-E sequence was confirmed using Sanger sequencing.

### 2.2. Cell Lines

Gene-modified K562 cells expressing membrane-bound IL-21 [34] were cultivated in complete RPMI-1640 medium (PanEco, Moscow, Russia) with 10% fetal bovine serum (FBS) (HyClone laboratories, Logan, UT, USA), 2 mM L-glutamine (PanEco), 1% sodium pyruvate (PanEco), and 1% antibiotic-antimycotic (Millipore Sigma, St. Louis, MO, USA). The original cells (K562-21) and the cells we obtained after genetic modification with HLA-E (K562-21E) were irradiated with 100 Gray and frozen for subsequent experiments.

Phoenix-Ampho cell line (ATCC, Manassas, VA, USA) was used as viral particle packaging cells and cultivated in a complete medium consisting of DMEM supplemented with 10% serum, 2 mM L-glutamine, 1% sodium pyruvate, and 1% antibiotic-antimycotic.

#### Retroviral Transduction

Transfection was performed in Phoenix Ampho cells using a Calcium phosphate buffer (Invitrogen, Waltham, MA, USA). Obtained retroviral particles were used to generate K562-21 cells expressing HLA-E (K562-21E). The resulting particles were filtered through a 0.45 nm PES filter (Millipore, Burlington, MA, USA) and concentrated by ultracentrifugation at 25,000× *g*. Transduction of K562-21 cells was carried out in plates treated with retronectin solution (Takara Bio, San Jose, CA, USA).

### 2.3. Peptide Synthesis

The following reagents were used: a polystyrene resin with a 2-chlorotrityl chloride handle, Fmoc-protected amino acids and HATU from GL Biochem Shanghai (Shanghai, China), TIS from Iris Biotech (Marktredwitz, Germany), 4-methylpiperidine from Mosinter Chemicals (Ningbo, China), trifluoroacetic acid from Solvay S.A. (Brussels, Belgium), and acetonitrile (gradient grade) from Biosolve (Dieuze, France). DCM was dried over CaH_2_ and refluxed before use. All other reagents and solvents were purchased from a local manufacturer and used without further purification.

Solid-phase syntheses of peptides were carried out using custom-made automated parallel peptide synthesizer based on the Gilson liquid handler. Fmoc strategy with HATU/DIPEA activation was applied. C-terminal amino acid was attached to the 2-chlorotrityl chloride-activated resin in the presence of DIPEA for 2 h. After that, all residues were coupled automatically by the following cycled procedure:

- PIP (30% solution in NMP, 25 mL for 1 g of resin), 2 × 10 min;
- DMF (20 mL for 1 g of resin), 5 × 5 min;
- Fmoc-AA(PG)-OH (8 eq., 0.5M solution in NMP)/HATU (8 eq., 0.5M solution in NMP)/DIPEA (16 eq.), 40 min;

DMF (25 mL for 1 g of resin), 5 × 5 min.

After the synthesis was completed, resin was washed with MeOH (30 mL for 1 g of resin, 5 × 5 min) and dried under reduced pressure.

For final deprotection of peptide, standard cleavage mixture was used (TFA/DTT/H_2_O/TIS 89/6/5/1) [35]. Dry peptidyl resin was suspended in cleavage mixture (15 mL for 1 g of resin) and was shaken for 2 h. After the filtration of the resin, diethyl ether (tenfold excess corresponding to cleavage mixture volume) was added and suspension was left to stand at −20 °C for couple of hours, centrifuged, washed 3 times with same amount of diethyl ether, and dried under reduced pressure.

### 2.4. NK Cell Isolation and Magnetic Separation of CD57^−^ Subset

Healthy individuals participated in the study, all of them provided verbal informed consent. To isolate the peripheral blood mononuclear cells (PBMC), blood samples were collected in EDTA-containing test tubes and centrifuged in a Ficoll gradient with 1.077 g/cm^3^ density (PanEco). NK cells isolated from PBMCs by negative magnetic separation (CD3^−^CD56^+^ cells) (Miltenyi Biotec, Bergisch Gladbach, Germany) with purity of >97% were applied for further culturing, phenotypic analysis, and subsequent separation of NK cells into subpopulations. To obtain CD57^−^ NK cells, CD57-positive magnetic cell separation was performed (Miltenyi Biotec, Bergisch Gladbach, Germany). The purity of CD57^−^ NK cell isolation was no less than 97%.

### 2.5. Incubation of HLA-E Modified Cells with Peptide

HLA-E expression on K562-21E feeder cells was stimulated by the cytomegalovirus peptide VMAPRTLFL. To do this, the cells were centrifuged at 200× *g* for 5 min, the supernatant was drained and resuspended in serum-free Opti-MEM medium (Thermo Fisher Scientific, Waltham, MA, USA) with 75, 150 or 300 µM VMAPRTLFL. Subsequently, the cells at concentrations from 2 × 10^5^ to 2 × 10^6^ cells/mL were incubated with the peptide for 1 to 24 h in a 24-well plate at 37 °C, 5% CO_2_.

### 2.6. HCMV IgG Detection

The HCMV-specific IgG levels in plasma samples of the healthy volunteers were measured using ELISA kit (D-1556, Vector-Best, Novosibirsk, Russia) according to the manufacturer’s protocol.

### 2.7. Cell Staining and Flow Cytometry Analysis

The following mouse anti-human fluorescent-labeled antibodies were used for surface cell staining: CD56-APC-Vio770 (clone REA196), CD57-VioBlue (clone TB03), CD107a-APC (clone REA792), HLA-DR-PE-Vio770 (clone REA805), IFNγ-PE (clone 45-15), KIR2DL1-APC (clone REA284), KIR2DL2/DL3-PE (clone DX27), NKG2C-VioBright (clone REA205), HLA-E-PE (clone REA1031) (Miltenyi Biotec, Bergisch Gladbach, Germany); CD56-BrilliantViolet 421 (clone 5.1 H11), CD57-PE (clone HNK-1), CD57-APC (clone HNK-1) (Sony Biotechnology, San Jose, CA, USA); NKG2C-PE (clone 134591) (R&D Systems, Minneapolis, MN, USA).

PBMC/NK cells were washed in the PBA staining buffer (PBS containing 0.5% BSA (bovine serum albumin) (PanEco, Moscow, Russia) and 0.01% sodium azide (PanEco, Moscow, Russia) and incubated with antibodies for 30 min on ice in a PBA then washed twice in the same buffer before measurement. Flow cytometry analysis was carried out on a MACSQuant 10 cytometer (Miltenyi Biotec, Bergisch Gladbach, Germany) equipped with 405 nm, 488 nm, and 635 nm lasers.

### 2.8. NK Cell Expansion

Bulk and CD57^-^negative NK cells were cultured in 96-well flat-bottom plates in DMEM and NK MACS medium (Miltenyi Biotec, Bergisch Gladbach, Germany) mixed in 1:1 proportion, supplemented with 10% fetal bovine serum (FSC) (HyClone Laboratories, Logan, UT, USA) and IL-2 = 100 units/mL, 2 mM L-glutamine (PanEco, Moscow, Russia), 1% sodium pyruvate (PanEco, Moscow, Russia), and 1% antibiotic-antimycotic (Millipore-Sigma). Irradiated K562-21 or K562-21E feeder cells were preincubated with peptides (as described above) and then coupled with NK cells at a ratio of NK:feeder cells = 1:1, and the media was refreshed every 2-3 days. Cells were incubated in 96-well flat-bottom plates and subsequently in 24-well plates at 37 °C and 5% CO_2_ concentration. Cells were subcultured to maintain an optimal concentration of 4–9 × 10^5^ cells/mL. To prevent excessive evaporation of the medium in the outer wells of a 96-well plate, cells were cultured in 60 central wells, and the periphery wells were filled with PBS. On days 6, 12, and 18, cells in the wells were counted, the expansion coefficients K_E_ (for cultures proliferated with feeder cells previously incubated with HCMV peptide and expressing HLA-E) and K_21_ (for control cultures, incubated with the same feeder cells that were incubated with the absence of any exogenous peptide) were calculated using the formula: K_X_ = N(t)/N(0), where N(t) is the number of cells N in the culture at time t.

### 2.9. Cell Proliferation

Freshly isolated NK cells were stained with 5 μM CFSE (Molecular Probes, Eugene, OR, USA) in 100 μL of PBS (PanEco, Moscow, Russia) and subsequently incubated for 15 min at 37 °C and protected from light. NK cells were washed three times in RPMI-1640 (PanEco, Moscow, Russia) supplemented with 5% FBS (Hyclone, Logan, UT, USA). NK cells were cultured with IL-2, IL-2+K562-21, or IL-2+K562-21E for 7 days according to the method described above.

### 2.10. Degranulation Assay

Degranulation assay was performed after 3 weeks of CD57^−^ NK cell cultures cultivation to estimate their natural cytotoxicity. Before stimulation, NK cells were incubated in DMEM with 10% FCS without IL-2 for 24 h. Then cells were stimulated with IL-2 = 500 units/mL for 16 h and K562 cells were added in a ratio 1:1 for 2.5 h in the presence of 10 mg/mL of brefeldin A (Sigma, Saint Louis, MO, USA) and CD107a-APC antibodies (clone REA 792, Miltenyi Biotec, Bergisch Gladbach, Germany). The degranulation level was determined by the proportion of NK cells expressing CD107a. Baseline levels of degranulated NK cells were determined in samples without K562 cells.

### 2.11. Cytokine-Dependent IFNγ Production Assay

IFNγ production by NK cells was determined in response to stimuli IL-12 and IL-18, together. Before stimulation, NK cells were incubated for 24 h without IL-2 and then stimulated with IL-12 (20 ng/mL) and IL-18 (20 ng/mL) for 20 h, including 4 h before measurement with 10 mg/mL brefeldin A. NK cells were fixed and stained with IFNγ-PE antibody (clone 45-15, Miltenyi Biotec, Bergisch Gladbach, Germany).

### 2.12. Statistical Analysis

Data were analyzed using GraphPadPrism (version 8.00, GraphPadSoftware, San Diego, CA, USA) and FlowJo VX (v10, FlowJo LLC, Ashland, OR, USA). The statistical significance of the obtained results was determined using a paired two-parameter Student *t*-test for data with a normal distribution; in other cases, the Mann–Whitney U test was used. For repeated measures, statistical significance was determined by a repeated measures ANOVA test, and multiple comparisons were carried out with a non-parametric Kruskal–Wallis test. Correlation analysis was done using Spearman correlation for nonparametric samples. Values *p* < 0.05 were taken as statistically significant: *—*p* < 0.05; **—*p* < 0.01; ***—*p* < 0.001, ****—*p* < 0.0001. Analysis results are presented as means ± standard deviation unless otherwise noted.

## 3. Results

### 3.1. Obtaining HLA-E-Expressing Feeder Cells on the Basis of K562 Cell Line and Their Characterization

The K562 cell line expressing IL-21 (K562-21) was genetically modified using retroviral particles containing HLA-E to obtain a new K562-21E cell line. The transduction efficiency was assessed by the level of GFP fluorescence. The GFP-positive cells were isolated by sorting.

**Figure 1:**
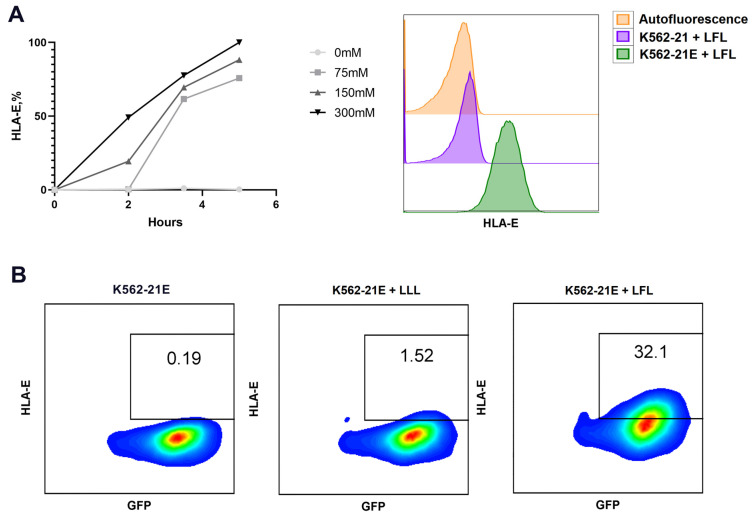
Characterization of obtained K562-21E cells. (**A**) Dependence of the HLA-E surface expression in the transduced K562-21E cells on the LFL peptide concentration (**left**). Representative histogram of HLA-E expression levels in K562-21 and K562-21E cells cultured with 300 µM of the LFL peptide (**right**). (**B**) Proportions of HLA-E-expressing cells in K562-21E cell cultures after incubation with LFL or LLL. For the experiment, irradiated K562-21E cells were used, which were subjected to a freezing and thawing cycle.

Since HLA-E only appears on the cell surface in the complex with a peptide [36], exogenous peptides were used as inductors of HLA-E expression. The VMAPRTLFL (LFL) and VMAPQSLLL (LLL) peptides (the latter served as a control due to its poor interaction with NKG2 receptors) were chosen according to previously published data and were synthesized [20,21]. Incubation of K562-21 cells with the LFL peptide did not result in an upregulation of surface HLA-E expression (Figure 1A); similarly, no HLA-E was detected after incubation with the LLL peptide.

HLA-E induction in response to the presentation of the LFL peptide in K562-21E cells required a screening of the incubation conditions. According to the primary report [21], the addition of 300 µM peptide increased HLA-E expression on the cell surface after 24 h of incubation in a serum-free medium. We observed that the level of HLA-E expression decreased with elevating concentrations of K562-21E cells due to increased cell death (Table 1). A concentration of 2 × 10^5^ cells/mL was chosen as optimal, corresponding to a maximum level in the surface HLA-E density without loss of cell viability. We found that the surface expression of HLA-E increased during the first hours of incubation and reached a maximum at the 5 h point (Figure 1A). Concentrations of the LFL peptide (75 μM, 150 μM, and 300 μM) induced different dynamics of HLA-E accumulation on K562-21E cells: 75 μM peptide induced HLA-E expression in 100% of K562-21E cells only in 3.5 h after adding the stimulus, whereas 150 and 300 μM of peptide provoked appearance of surface HLA-E after 2 h (Table 1). Further incubation was associated with an increase in the density of HLA-E after 3.5 and 5 h and did not lead to an increase in HLA-E expression during longer cultivation (Table 1).

The effects of gamma irradiation and repetitive freeze–thaw cycles on K562-21E cells’ ability to present HLA-E were tested after incubation with LFL and LLL peptides for 5 h. The addition of the LFL peptide induced HLA-E surface expression in thawed cells, although it was not as pronounced as in live non-irradiated cells. Meanwhile, the control LLL peptide still did not induce HLA-E surface expression (Figure 1B).

### 3.2. HLA-E-Presenting K562-21E Cells Induce Active NK Cell Proliferation

**Figure 2:**
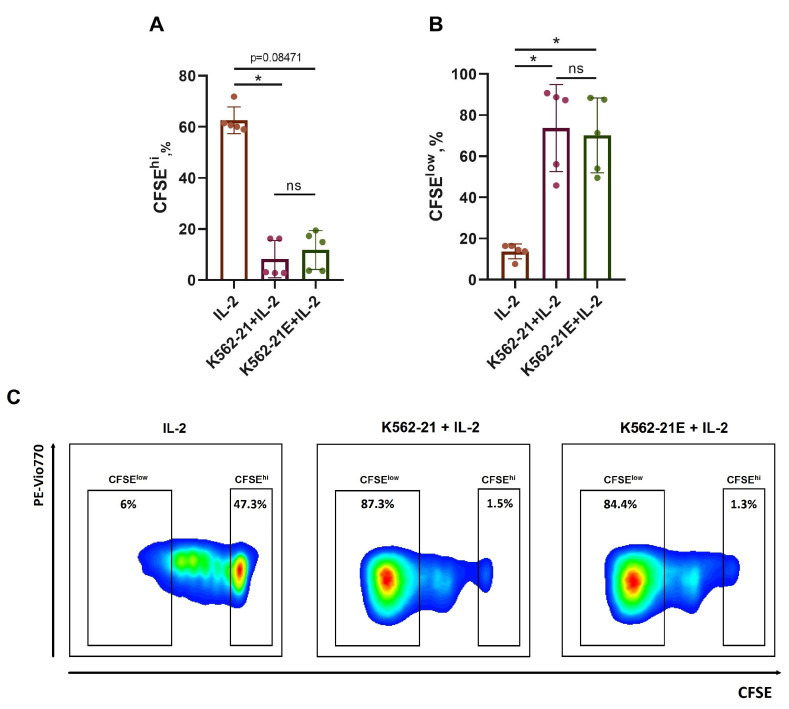
Proliferative activity of bulk NK cell cultures under IL-2 stimulation in combination with K562-21 or K562-21E feeder cells. (**A**) The proportion of CFSE^hi^ NK cells that corresponds to non-dividing cells in response to stimulation. N = 5. (**B**) The proportion of CFSE^low^ NK cells observed in cells divided six or more times in response to the stimulus. N = 5. (**C**) The level of intercellular CFSE content in CFSE-stained bulk cultures of NK cells after IL-2 stimulation, both without or in combination with K562-21 or K562-21E feeder cells presenting LFL peptides in complex with HLA-E. Cultivation time t = 7 days. Statistical analysis was performed using a nonparametric Kruskal–Wallis test for multiple comparisons (* *p* < 0.05, ns—not significant), means ± SD are shown.

To confirm the ability of K562-21E cell cultures to activate NK cells, the proliferative activity of bulk CFSE-stained NK cells was assessed after being stimulated for 7 days with K562-21E+IL-2 and compared to K562-21+IL-2 stimulation (Figure 2). In both variants, the LFL peptide was added. The stimulation with 100 U/mL IL-2 without feeder cells was used as a control. There were no differences in the proportion of actively proliferating (CFSE^low^) and nondividing (CFSE^hi^) NK cells between samples with K562-21 and K562-21E stimulus, while a significantly lower proportion of proliferating NK cells was demonstrated in the control (Figure 2A–C). Of note, there were no differences in CD16 (ADCC receptor) and HLA-DR (late activation marker of NK cells) expression levels between the cultures stimulated with K562-21 and K562-21E cells (Figure S1). Thus, the modification with HLA-E did not affect the feeder traits of the K562-21E cells.

### 3.3. Analysis of CD57-Negative NK Cell Expansion Induced with the Use of HLA-E-Expressing K562 Feeder Cells

#### 3.3.1. Twelve-Day Expansion of CD57^−^ NK Cells Is Greater upon Presentation of the HLA-E/LFL Complex, Regardless of HCMV Serological Status

To compare the direct effect of LFL in complex with HLA-E on NK cells, NK cell expansion rates were measured under IL-2 stimulation in combination with K562-21E feeder cells, both in the presence of or without LFL. We used CD57-negative cells, characterized by a higher proliferative potential compared to the more highly differentiated CD57^+^ NK cells. A pool of less mature NKG2C^+^ cells capable of recognizing HCMV peptides exposed in HLA-E was also observed in a CD57^−^ population of NK cells, from both HCMV-seropositive and seronegative individuals. In addition, CD57-negative NK cells presumably contain precursor subsets of HCMV-associated adaptive NK cells bearing the CD56^dim^CD57^−^NKG2C^+^ phenotype. Such cells were described by having additional phenotypic similarities with highly differentiated adaptive CD57^+^NKG2C^+^NKG2A^−^NK cells [32]. In this study, the data from seven healthy volunteers (D1–D7) of different genders and ages with known HCMV-specific IgG serological titers were obtained. As a result, two highly HCMV-seropositive individuals, three with low levels of anti-HCMV IgG, and two seronegative individuals were included in the study (Table 2).

The effect of a transient presentation of the LFL peptide on the proliferation of a heterogeneous pool of CD57^−^ NK cells was investigated. Freshly isolated by negative magnetic separation, CD57^−^ NK cells were either cultured with IL-2 and K562-21E feeder cells bearing the HLA-E/LFL complex, or with the same feeder cells without the LFL-peptide, used as a control.

Ex vivo NK cell phenotypic characteristics were evaluated for each donor. The expression levels of the NKG2A and NKG2C receptors recognizing HLA-E, KIR3DL2/3 and KIR2DL1 inhibitory receptors, the HLA-DR activation marker, and the proportion of potential HCMV-associated adaptive NK cells were measured (Figure S2). The expansion coefficients of the obtained cultures, K_E_—the expansion coefficient of NK cell cultures stimulated by K562-21E cells loaded with LFL— and K_21_—the expansion coefficient of control NK cell cultures, stimulated by K562-21E cells without adding LFL—were analyzed 6, 12, and 18 days after pulse stimulation with the feeder cells. We also evaluated the ratio of the expansion coefficients of these cultures, K_E_/K_21_. This relative expansion coefficient, K_E_/K_21,_ is greater than one when NK cells exhibit greater expansion in response to HLA-E/LFL presentation than they do without the LFL presentation.

**Figure 3:**
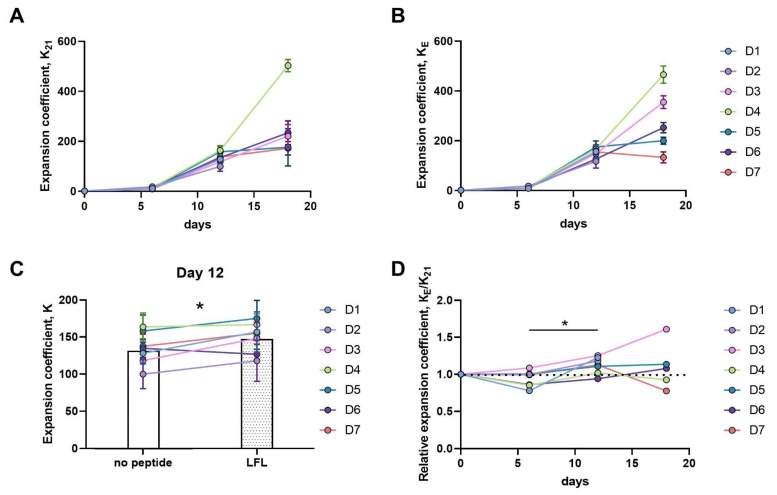
Proliferative activity of CD57^−^ NK cells in response to IL-2 stimulation in combination with K562-21E feeder cells with or without LFL peptide. Number of donors n = 7, data for each donor (D1–D7) is indicated by seven different colors. (**A**) Expansion coefficient of CD57^−^ NK cells in response to stimulation by IL-2 and K562-21E feeder cells that were not incubated with LFL peptide, K_21_ = N(t)/N(0), where N(t) is the number of cells in culture at time t; (**B**) Expansion coefficient of CD57^−^ NK cells in response to stimulation by IL-2 and K562-21E feeder cells presenting the LFL peptide by HLA-E, K_E_ = N(t)/N(0), where N(t) is the number of cells in culture at time t; (**C**) Expansion coefficient of CD57^−^ NK cells on day 12 of stimulation by IL-2 and K562-21E feeder cells presenting and not presenting the LFL peptide in HLA-E. Statistical analysis was performed using paired *t*-test (* *p* < 0.05); means ± SD are shown; (**D**) The ratio of expansion coefficients of CD57^−^ NK cell cultures obtained by stimulation with feeder cells K562-21E, where K_E_/K_21_ = K(K562-21E + LFL)/K(K562-21E without peptide incubation). Dashed line corresponds to K_E_/K_21_ = 1. Statistical analysis was performed using a repeated measures ANOVA test (* *p* < 0.05); means ± SD are shown.

More than a hundredfold increase in the number of cells was observed for all the donors after 12 days of cultivation (Figure 3A,B). At this time point, a higher expansion coefficient was observed in CD57^−^ NK cell cultures when the LFL peptide was presented (Figure 3C). In comparison to day 6, an increase in the K_E_/K_21_ relative expansion coefficient was observed, which indicated that the peptide presentation induced a more intensive expansion of CD57^−^ NK cells, regardless of the donor’s serological status (Figure 3D). However, on day 18, pronounced donor-dependent variations in the proliferation rates of NK cultures were observed. NK cells of two highly HCMV-seropositive (D3, D6) and one HCMV-seronegative (D5) donor proliferated better on day 18 after the HLA-E/LFL presentation, whereas the reverse trend was revealed in NK cells obtained from the blood of HCMV-seropositive individuals with a moderate titer of IgG to HCMV (D4, D7) (Figure 3D). Notably, different K_E_/K_21_ dynamics were shown in the NK cell cultures of D3 and D6 donors. The CD57^−^NK cells of D3 demonstrated more intense proliferative activity during the entire observation period when stimulated by LFL-presenting feeder cells. By day 18, in the D3 NK cell cultures exposed to HLA-E/LFL, 60% more cells were observed compared to the control. In the NK cell cultures of D6 (a highly HCMV-seropositive individual), less intensive proliferation on the 6th and 12th days of cultivation was observed in response to the HLA-E/LFL presentation compared to cells cultured without the peptide. However, on day 18, these HLA-E/LFL-stimulated cells performed a higher expansion coefficient compared to the control cultures (Figure 3D).

#### 3.3.2. An Increase in the Proportion of CD56^bright^HLA-DR^+^ NK Cell Subset Ex Vivo Is Associated with More Intense Proliferation of NK Cells in Response to the LFL Peptide Presentation by K562-21E Feeder Cells

**Figure 4:**
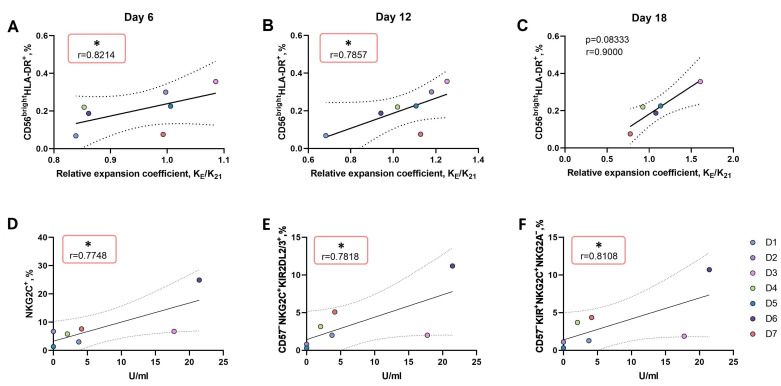
Correlations of ex vivo CD57^−^ NK cell phenotypes with the immune response to HCMV infection and with the proliferative activity of CD57^−^ NK cells during LFL peptide presentation by K562-21E feeder cells. (**A**–**C**) Correlation between the proportion of CD56^bright^HLA-DR^+^ NK cells ex vivo and the relative expansion coefficient KE/K21 on days 6 (**A**), 12 (**B**), and 18 (**C**), KE—expansion coefficient in cultures stimulated by IL-2 and K562-21E cells presenting LFL peptide, K21—expansion coefficient in cultures stimulated by IL-2 and K562-21E feeder cells not presenting LFL peptide, n = 7. (**D**–**F**) Correlations between the phenotype of CD57^−^ NK cells ex vivo and serological titer to HCMV in donors’ plasma; (**D**) Correlation between the proportion of NKG2C^+^ NK cells ex vivo and the titer of antibodies to HCMV in donors’ plasma; (**E**) Correlation between the proportion of CD57^−^NKG2C^+^KIR2DL2/3^+^ NK cells ex vivo and the titer of antibodies to HCMV in donors’ plasma; (**F**) Correlation between the proportion of CD57^−^NKG2C^+^KIR2DL2/3^+^NKG2A^−^ NK cells ex vivo and the titer of antibodies to HCMV in donors’ plasma; Correlation analysis was done using Spearman correlation for nonparametric samples, * *p* < 0.05 was considered statistically significant. Solid line in each figure represents a linear regression line, 95% confidence interval is bounded by two dashed lines.

Since we identified donor-dependent differences in the proliferative response of CD57^−^ NK cells to IL-2 in combination with K562-21E feeder cells presenting the LFL peptide, we searched for correlations between the phenotypic traits of CD57^−^ NK cells ex vivo and their expansion coefficients on day 12, when a more pronounced expansion was observed in the NK cell cultures to which the LFL peptide was presented. The total ex vivo phenotype data, along with the IgG titer and expansion coefficient, were used to identify the main coordinations between donors via principal component analysis (Figure S3) for further direct demonstration of exact correlations. The only factor which directly correlated with the relative expansion coefficient K_E_/K_21_ throughout the entire cultivation period was the proportion of CD56^bright^HLA-DR^+^ NK cells (Figure 4A–C). At the same time, negative correlations were found between the expansion rates under HLA-E/LFL stimulation and the ex vivo proportion of cells bearing markers of greater maturity: NKG2C^+^NKG2A^+^, CD56^bright^NKG2C^+^, and CD56^bright^KIR2DL2/3^+^NKG2C^+^NKG2A^−^. In cultures to which the peptide was not presented, there was no relationship between the proliferation intensity and the proportion of potential precursors of adaptive NK cells ex vivo (Figures S4 and S5).

We also tried to find relationships between the ex vivo NK cell phenotype and the HCMV-specific IgG serum titer. A previously described relationship between the size of the NKG2C^+^ cell fraction and the increase in HCMV-IgG titer in the individual’s plasma was confirmed [18,37] (Figure 4D). In addition, a direct relationship was revealed between the anti-HCMV IgG titer and the percentages of two subsets of differentiated NKG2C^+^ cells in the total NK cell population ex vivo: licensed CD57^−^KIR2DL2/3^+^NKG2C^+^ cells capable of accumulating cytotoxic granules (Figure 4E) and potential precursors of adaptive NK cells with the CD57^−^KIR2DL2/3^+^NKG2C^+^NKG2A^−^ phenotype (Figure 4F). These positive correlations identified a typical pattern in the coordination of the immune response to HCMV in our cohort, despite the small sample size.

#### 3.3.3. In CD57^−^ NK Cell Cultures Actively Proliferating in Response to the Presentation of the HCMV Peptide, a High Proportion of KIR2DL2/3^+^NKG2C^+^NKG2A^−^ Presumable Progenitors of Adaptive NK Cells was Observed

**Figure 5:**
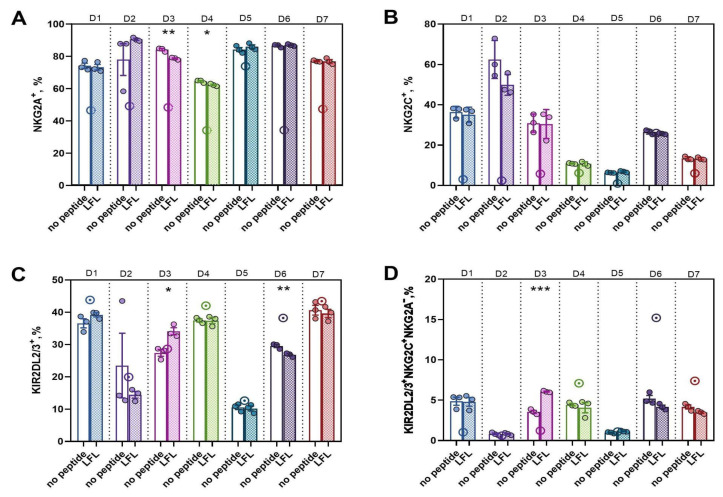
Phenotype of NK cell cultures obtained from CD57^−^NK cells after their stimulation with IL-2 and K562-21E feeder cells with or without the addition of the LFL peptide. The proportion of NKG2A^+^ NK cells (**A**), NKG2C^+^ NK cells (**B**), KIR2DL2/3^+^ NK cells (**C**), and KIR2DL2/3^+^NKG2C^+^NKG2A^−^ (**D**) in CD57− NK cell cultures. Large circled dots represent the expression level of the markers in the ex vivo NK cell CD57^−^ population. Small colored dots represent the expression level of the markers in the cultures after stimulation. Stimulation time t = 14 days, number of donors n = 7, data for each donor (D1–D7) is indicated by seven different colors. Statistical analysis was performed using an unpaired *t*-test (* *p* < 0.05, ** *p* < 0.01, *** *p* < 0.005); means ± SD are shown.

To assess how a single time stimulation by the LFL-loaded K562-21E cells affects the CD57^−^ NK cell phenotype, the surface expressions of NKG2A, NKG2C, KIR2DL1/3, CD57, and KIR2DL1 markers in the obtained cultures were measured by flow cytometry after cultivation for two weeks. In all NK cell cultures, regardless of the method of stimulation, an increased proportion of NKG2A^+^ cells was observed after expansion compared to the ex vivo level (Figure 5A). Interestingly, in the CD57^−^ NK cell cultures of HCMV highly seropositive donor D3, intensively proliferating in response to HLA-E/LFL presentation, a lower proportion of NKG2A^+^ cells, as well as higher proportions of KIR2DL2/3^+^ cells and cells with the CD57^−^KIR2DL2/3^+^NKG2C^+^NKG2A^−^ phenotype of potential precursors of adaptive NK cells were observed compared to the control cultures (Figure 5A,C,D). An increase in the proportion of NKG2C^+^ cells and KIR2DL2/3^+^NKG2C^+^ cells was observed in CD57^−^ NK cell cultures of all donors with the exception of D6 (Figure S6), whose NK cells demonstrated the most differentiated phenotype (Figure S2). Also, regardless of the stimulation method, a decrease in the proportion of KIR2DL2/3^+^ cells was more pronounced in the NK cell cultures of all donors, with the exception of D3, and a loss of this marker expression was observed in D6’s cultures when stimulated by feeder cells with HLA-E/LFL (Figure 5C).

#### 3.3.4. Active Proliferation of CD57^−^ NK Cells in Response to the LFL Peptide Presentation Is Accompanied by Positive Functional Shifts

**Figure 6:**
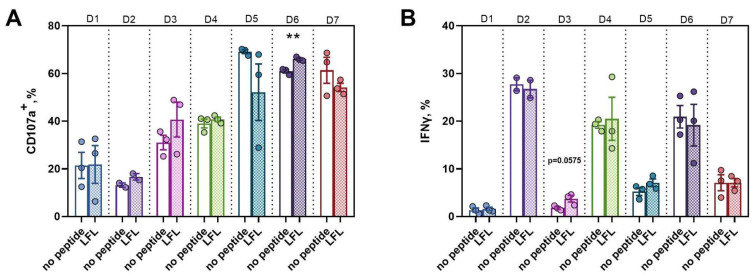
Functional activity of cultures of CD57^−^NK cells after stimulation with IL-2 and K562-21E feeder cells, presenting and not presenting the LFL peptide as part of HLA-E. (**A**) The proportion of degranulated NK cells after incubation with target cells K562 CD107a^+^; (**B**) The proportion of NK cells producing IFNγ in response to stimulation with IL-12 and IL-18. Number of donors n = 7, data for each donor (D1-D7) is indicated by seven different colors, dots represent the proportion of functionally active cells in each culture. t = 18 days. Statistical analysis was performed using an unpaired *t*-test (** *p* < 0.01).

Taking into account that the presentation of LFL by K562-21E feeder cells can affect both the phenotype of proliferating NK cells and their functional activities, we assessed the functional potential of the CD57^−^ NK cell cultures on day 18 of cultivation. The natural cytotoxicity of the NK cell cultures was assessed by the proportion of degranulating NK cells in response to K562 target cells. All obtained cultures exhibited cytotoxic activity regardless of the stimulation method or donor’s HCMV serological status. However, the CD57^−^ NK cell cultures of donor D6, distinguished by a high HCMV-IgG IgG titer and a late response to the presentation of the LFL peptide by feeder cells, had the greatest cytotoxic activity (Figure 6A).

Also, on the ^1^8th day of cultivation, the production of IFNγ by the CD57^−^ NK cell cultures in response to stimulation by the proinflammatory cytokines IL-12 and IL-18 was assessed. Production levels varied widely between donors. The CD57^−^ NK cell cultures of donor D3 that were presented with the LFL peptide tended to have a higher proportion of IFNγ-producing cells compared with control cultures without peptide (Figure 6B).

## 4. Discussion

It is well known that NKG2C expression is associated with the generation of a pool of adaptive NK cells, which are essential in the control of HCMV infections [21,38,39]. Yet, the role of NKG2C-mediated interactions in the development of adaptive NK cells in humans is still not well understood. While the presence of NKG2C^+^ NK cells has been identified in the majority of HCMV-seropositive individuals, its percentage exhibits considerable variation (from 2% to 70%). Additionally, a minor subset of NKG2C^+^ NK cells can also be observed in HCMV seronegative individuals [32]. The reasons for such a significant heterogeneity, as well as its clinical significance, need to be investigated. This heterogeneity may be caused in part by different cytokine environments, under which the NKG2C^+^ NK cell subset develops. In particular, proinflammatory cytokines such as IL-12 are assumed to be necessary for the NKG2C^+^ adaptive NK cell formation [40]. The number of gene copies encoding NKG2C also affects the number of NKG2C^+^ NK cells [38]. Recently, it was shown that, among elderly individuals, a significant fraction of NKG2C^+^CD57^+^ NK cells in peripheral blood could be observed even for HCMV-seronegative persons. Interestingly, in this HCMV-seronegative group, a positive correlation was revealed between the proportion of NKG2C^+^CD57^+^ NK cells and age, whereas no such correlation was found in HCMV-seropositive donors [41]. Notably, the NKG2C receptor is not obligatory for the development of an adaptive-like NK cell response: in KLRC2^del/del^ individuals infected with HCMV, a special subset of adaptive NK cells expressing activating KIRs can be found [42].

A number of studies have demonstrated an essential role of HLA-E-associated peptides in the selective NKG2C-mediated recognition and activation of NK cells [43]. Prolonged cultivation of NK cells with a cell line expressing HLA-E [21] or with an HLA-E trimer [44] loaded with the VMAPRTLFL peptide (LFL in this work), led to the proliferation of NK cells, an increase in their functional activity, and the acquisition of the “adaptive” phenotype [20]. Several methods exist to enhance the peptide presentation in the HLA complex; in this work, the method of HLA-E loading by exogenous peptides was chosen. This approach is more practically applicable compared to the method involving simultaneous cell transduction with two transgenes: the HLA molecule and the leader peptide sequence [44]. The permanent expression of the leader peptide will lead to the stable surface expression of the HLA-E/peptide complex, which, in turn, might cause NK cell overactivation. On the contrary, the addition of an exogenous peptide to K562-IL21E cells seems to promote more physiological settings for the interaction of the HLA-E/peptide complex and the NKG2C receptor. In addition, unlike HLA-E^+^ feeder cell lines stably expressing HCMV peptide, the K562-21E cells produced in this work were able to present a wider variety of stimuli in the context of HLA-E. Such a model allows to expand the research in studying NK cell immune reactions and differentiation in response to other peptides, for example, derived from SARS-CoV2 or other pathogens [45,46].

The activation of NK cells by K562-21E cells loaded with the LFL peptide allows to induce a secondary response of HCMV-associated memory NK cells and to accumulate HCMV-specific cells. Moreover, this in vitro model of NK cell activation may help to clarify the mechanism of initial NKG2C induction in both HCMV-seropositive and seronegative individuals and the role of NKG2C expression in lower-differentiated CD56^bright^ NK cells, as well as the relationship of the NKG2C expression with the formation of an adaptive NKG2C^+^CD57^+^ NK cell subset.

In this work, we assessed the influence of obtained K562-21E feeder cells presenting HLA-E/LFL on CD57-negative NK cell proliferation, phenotype, and functional activity. We have previously shown that CD57^−^CD56^dim^NKG2C^+^ NK cells share certain phenotypic features with the adaptive-like CD57^+^NKG2C^+^ cells. These HCMV-specific cells are characterized by a high lifespan and antibody-dependent cytotoxicity but low proliferative capacities [32]. CD57^−^CD56^dim^NKG2C^+^ NK cells perform transitional stages in the process of NK cell differentiation and, at the same time, are characterized by a higher proliferative potential and survival. We have also revealed that CD57^−^ NK cells expressing NKG2C and KIR2DL2/3 are more susceptible to retroviral transduction compared to less differentiated NKG2C^−^KIR2DL2/3^−^ cells, which makes CD57^−^NKG2C^+^KIR2DL2/3^+^ cells a promising source for genetic modification and further therapeutic application [47]. Therefore, approaches that facilitate the accumulation of CD57^−^NKG2C^+^KIR2DL2/3^+^ cells are required for generating large numbers of NK cells without losing NKG2C and the inhibitory receptors KIR2DL2/3, which mark cytotoxic mature cells with an increased accumulation of cytotoxic vesicles [48]. The use of K562-21E feeder cells presenting the HCMV peptide for NK cell stimulation may represent one such approach.

In this work, we have shown that, regardless of the serological status of an individual, stimulation by K562-21E feeder cells presenting LFL at the initial stages of cultivation can induce a more rapid expansion in CD57^−^ NK cell cultures compared to control cultures. Thus, apparently, the expression of NKG2C by non-specialized naive NK cells may enhance their proliferative response during co-incubation with HLA-E-expressing cells loaded with LFL. Throughout the entire observation period, we defined a positive correlation between the relative expansion coefficient K_E_/K_21_ and the proportion of activated CD56^bright^HLA-DR^+^ cells (Figure 4D–F). HLA-DR is a marker of NK cells’ late activation [49]; its expression is associated with high proliferative rates and increased production of IFNγ [50]. HLA-DR is stably expressed on the surface of adaptive NKG2C^+^ NK cells, independently of other activation markers [51,52]. In inflammatory conditions, a high proportion of HLA-DR^+^ NK cells is often observed [53]. Moreover, it has been shown that during HCMV infection, HLA-DR^+^ NK cells are able to present viral antigens to CD4^+^ T cells and promote the development of an adaptive response [51]. A high proportion of CD56^bright^HLA-DR^+^ cells may be associated with the acute phase of cytomegalovirus infection and, as a consequence, could lead to a higher susceptibility of NK cells to stimulation by K562-21E feeder cells presenting the LFL peptide. Also, we have previously shown that the proliferation of poorly differentiated CD57^−^NKG2C^−^ NK cells with the K562-21 feeder cells induced the expression of NKG2C de novo [32]. The induction of NKG2C surface expression on CD56^bright^HLA-DR^+^ cells may lead to the subsequent recognition of the LFL peptide and, therefore, enhance the intracellular activating signal, resulting in a more intense proliferation in cultures incubated with feeder cells presenting the LFL peptide as part of HLA-E.

The most pronounced response of CD56^bright^ NK cells to stimulation can be associated with the expression of high affinity IL-2 receptor’s subunit on the CD56^bright^ NK cells and their major sensitivity towards soluble stimuli [54]. During differentiation, the full-sized IL-2 receptor, including the alfa chain, ceases to be expressed and is replaced by a receptor with medium affinity resulting in decreasing sensitivity to IL-2 stimulation. Furthermore, we also detected a negative correlation between the expansion rates under HLA-E/LFL stimulation and the proportion of cells at a later stage of maturation, including CD56^bright^KIR2DL2/3^+^NKG2C^+^NKG2A^−^ cells (Figure S4). NK cells of this subset are phenotypically similar to the progenitors of adaptive NK cells and may increase due to NK cells’ expansion in response to viral infection. Usually, this process is accompanied by the accumulation of dysfunctional mitochondria and mitochondrial-associated ROS, leading to apoptosis. Only a few of the expanded NK cells survive via the reactivation of autophagy and contribute to the pool of resting memory cells and are able to productively proliferate in response to HCMV antigen presentation [55].

With the longer cultivation of CD57^−^ NK cells (t = 18 days), a contribution to the clonal proliferation of some more differentiated CD56^dim^ cells in response to the LFL peptide presentation by K562-21E cells apparently occurred. In our work, the most pronounced response to HLA-E/LFL presentation was observed in the NK cell cultures derived from a highly HCMV-seropositive donor. Active proliferation was accompanied by the accumulation of CD57^−^KIR2DL2/3^+^NKG2C^+^NKG2A^−^ cells, which share properties with genuine adaptive NK cells [32]. We also detected a tendency towards a greater proportion of IFNγ-producing cells in cultures stimulated by HLA-E/LFL. Previously, it has been shown by another group that IFNγ mRNA levels increase under stimulation by LFL-presenting cells [21]. Therefore, K562-21E cells loaded with LFL could be used for the accumulation of adaptive cell progenitors with promoted functional properties in vitro. It suggests that there could be a connection between the HCMV-IgG titer, reflecting an HCMV infection stage, and sensitivity towards LFL stimulus. But what is remarkable is that the NK cells of one of the HCMV-seronegative donors also tended to expand more under HLA-E/LFL stimulus, even though these LFL-stimulated cells did not differ in phenotype or functional traits from the control cultures. Therefore, K562-21E feeder cells loaded with LFL peptide could be successfully exploited in order to accumulate NK cells in vitro. However, NK cell cultures of a HCMV-seronegative donor induced by both stimuli shared the same phenotype and functional properties. It can be assumed that LFL-presenting feeder cells can induce a primary-like response in CD57^−^NK cells to a simulated infection, during which the expression of HLA-G could be induced with the release of the corresponding leader peptide to the surface in the context of HLA-E. It is also quite possible that during the proliferation of NK cells obtained from the blood of HCMV-seropositive donors, we observe not only a secondary response of adaptive cells and cells with the phenotype of their predecessors, but a simultaneous mixed secondary and primary response of NK cells to HCMV, during which new adaptive memory cells can be formed.

Thus, the K562-21E cells obtained by retroviral transduction by the HLA-E gene have great potential, both in the field of practical applications inducing the accumulation of effector cells for cellular therapy, and in the field of fundamental studies investigating the processes underlying NK cell responses to activation with HCMV peptides derived from the UL40 protein. We showed that K562-21E cells, loaded with the HCMV peptide, are able to induce the proliferation of CD57-negative NK cells independently from the serological HCMV status of the donor. Nonetheless, the most pronounced proliferative response to the LFL peptide presentation and accumulation of cells with the phenotype of adaptive cells’ progenitors was observed in NK cell cultures, derived from the donor with a high HCMV-specific antibody titer.

## Figures and Tables

**Table 1:** Effect of stimulation conditions of K562-21E cells with the LFL peptide on the induction of HLA-E expression. The increase in HLA-E expression was calculated as the ratio of the geometric mean fluorescence (MFI) of the sample to the geometric mean fluorescence of the control without peptide.

K562-21E, 10^3^/mL	Peptide Concentration, µM	Increase in the Level of HLA-E Expression Compared to Control, Arbitrary Units					Cell Mortality during 24 h Incubation, %
		1 h	2 h	3.5 h	5 h	24 h	
200	0	1	1	1	1	1	
	75	1.01	1.01	2.59	3.77		
	150	1.02	2.61	2.8	4.37		
	300	1.02	3.93	3.03	5.01	3.58	11.8
500	300					3.44	28
700						3.31	33
2000						1.15	70

**Table 2:** The demographic donors’ characteristics and HCMV serological statuses.

Donor Number	Age	Gender	HCMV Titer Range (U/mL)
1	24	Male	3.7
2	25	Female	0
3	59	Female	17.8
4	40	Male	2.1
5	26	Male	0
6	64	Female	21.4
7	29	Female	4.1

## Data Availability

The data supporting the conclusions of this article will be made available by the authors upon request.

## Supplementary Materials

The following supporting information can be downloaded at https://www.mdpi.com/article/10.3390/pharmaceutics16010133/s1. Figure S1: The proportion of the NK cells’ functional activity markers in the bulk cultures of NK cells after IL-2 stimulation without or with K562-21 or K562-21E feeder cells presenting LFL peptide. Cultivation time t = 7 days. (A) The proportion of CD16^+^ NK cells (B), proportion of HLA-DR^+^ NK cells. Figure S2: The ex vivo analysis of NK cells phenotype by flow cytometry. Cell populations, in which the proportions of NK cell subsets were measured, are indicated above each graphic. (A) The proportion of NK cells in PBMC; (B) The proportions of CD56^bright^, CD57^+^, CD57^+^NKG2C^+^NKG2A^−^, and CD57^+^NKG2C^+^NKG2A^+^ subsets in population of NK cells. (C) The proportions of NKG2C^+^, NKG2A^+^, KIR2DL2/3^+^ KIR2DL1^+^, KIR2DL1^+^KIR2DL2/3^+^ KIR2DS1^+^, KIR2DS4^+^, NKG2A^+^NKG2C^+^, KIR2DL2/3^+^NKG2C^+^NKG2A^−^, KIR2DL2/3^+^NKG2C^+^NKG2A^+^, HLA-DR^+^, CD56^dim^HLA-DR^+^, CD56^bright^HLA-DR^+^, CD56^bright^NKG2C^+^, and CD56^bright^KIR2DL2/3^+^NKG2C^+^NKG2A^−^ subsets in population of NK cells. n = 7. Figure S3: Principal component biplot showing relationship among seven donors (points) and their phenotype characteristic, expansion rate, IgG titer (vectors). Dotted vertical and horizontal lines indicate points where the PC1 and PC2 axes had respective values of zero. Figure S4: Correlations between the phenotype of CD57^−^ NK *cells* ex vivo and their proliferative activity in response to IL-2 stimulation combined with K562-21E feeder cells presenting and not presenting HCMV peptide LFL. (A) Correlation between the proportion of NKG2C^+^NKG2A^+^ NK cells ex vivo and the expansion coefficient in CD57^−^ cultures of NK cells to which the LFL peptide was presented by K562-21E feeder cells. (B) Correlation between the proportion of NKG2C^+^NKG2A^+^ NK cells ex vivo and the expansion coefficient in CD57^−^ cultures of NK cells stimulated by K562-21E feeder cells without LFL peptide presentation. (C) Correlation between the proportion of CD56^bright^NKG2C^+^ NK cells ex vivo and the expansion coefficient in CD57^−^ cultures of NK cells to which the LFL peptide was presented by K562-21E feeder cells. (D) Correlation between the proportion of CD56^bright^NKG2C^+^ NK cells ex vivo and the expansion coefficient in CD57^−^ cultures of NK cells stimulated by K562-21E feeder cells without LFL peptide presentation. (E) Correlation between the proportion of CD56^bright^KIR2DL2/3^+^NKG2C^+^NKG2A^+^ NK cells ex vivo and the expansion coefficient in CD57^−^ cultures of NK cells to which the LFL peptide was presented by K562-21E feeder cells. (F) Correlation between the proportion of CD56^bright^KIR2DL2/3^+^NKG2C^+^NKG2A^+^ NK cells ex vivo and the expansion coefficient in CD57^−^ cultures of NK cells stimulated by K562-21E feeder cells without LFL peptide presentation. Correlation analysis was done using Spearman correlation for nonparametric samples, *p* < 0,05 was considered statistically significant. Figure S5: Correlations between the phenotype of CD57^−^NK cells ex vivo and their proliferative activity after IL-2 stimulation in combination with K562-21E cells presenting and not presenting HCMV peptide (LFL). (A) Spearman correlation between the proportion of CD56^bright^KIR2DS4^+^ NK cells ex vivo and the expansion coefficient in the CD57−NK cells cultures to which the HCMV peptide was presented once by K562-21E feeder cells, t = 12 days, n = 7. (B) Spearman correlation between the proportion of CD56^bright^KIR2DS4^+^ NK cells ex vivo and the expansion ratio in cultures of CD57− NK cells stimulated with K562-21E feeder cells without HCMV peptide, t = 12 days, n = 7. (C,D) Spearman correlation between the proportion of CD57^−^KIR2DL1^+^KIR2DL2/3^+^ NK cells ex vivo and the relative expansion coefficient, K_E_/K_21_ on days 6 (C) and 12 (D) of culture. K_E_ is the expansion coefficient in cultures, stimulated with IL-2 and K562-21E feeder cells presenting the HCMV peptide, K_21_ is the expansion coefficient in cultures stimulated with IL-2 and K562-21E feeder cells not presenting the HCMV peptide, n = 7. Figure S6: Phenotype of NK cell cultures obtained from CD57^−^ NK cells after their stimulation with IL-2 and K562-21E feeder cells with and without the LFL peptide. (A) Proportion of NKG2C^+^NKG2A^+^ NK cells, (B) KIR2DL2/3^+^NKG2C^+^ NK cells, and (C) KIR2DL2/3^+^NKG2C^+^NKG2A^+^ NK cells in CD57^−^ NK cell cultures. Large dots represent the expression level of the markers in the ex vivo CD57^−^ NK cell population. Stimulation time t = 14 days, N = 7. Means ± SD are shown.
