# Supplementary figures and images for: Obtaining Gene-Modified HLA-E-Expressing Feeder Cells for Stimulation of Natural Killer Cells
Source: Pharmaceutics. 2024 Jan 19;16(1):133. doi: 10.3390/pharmaceutics16010133 (PMC10818548; doi:10.3390/pharmaceutics16010133)

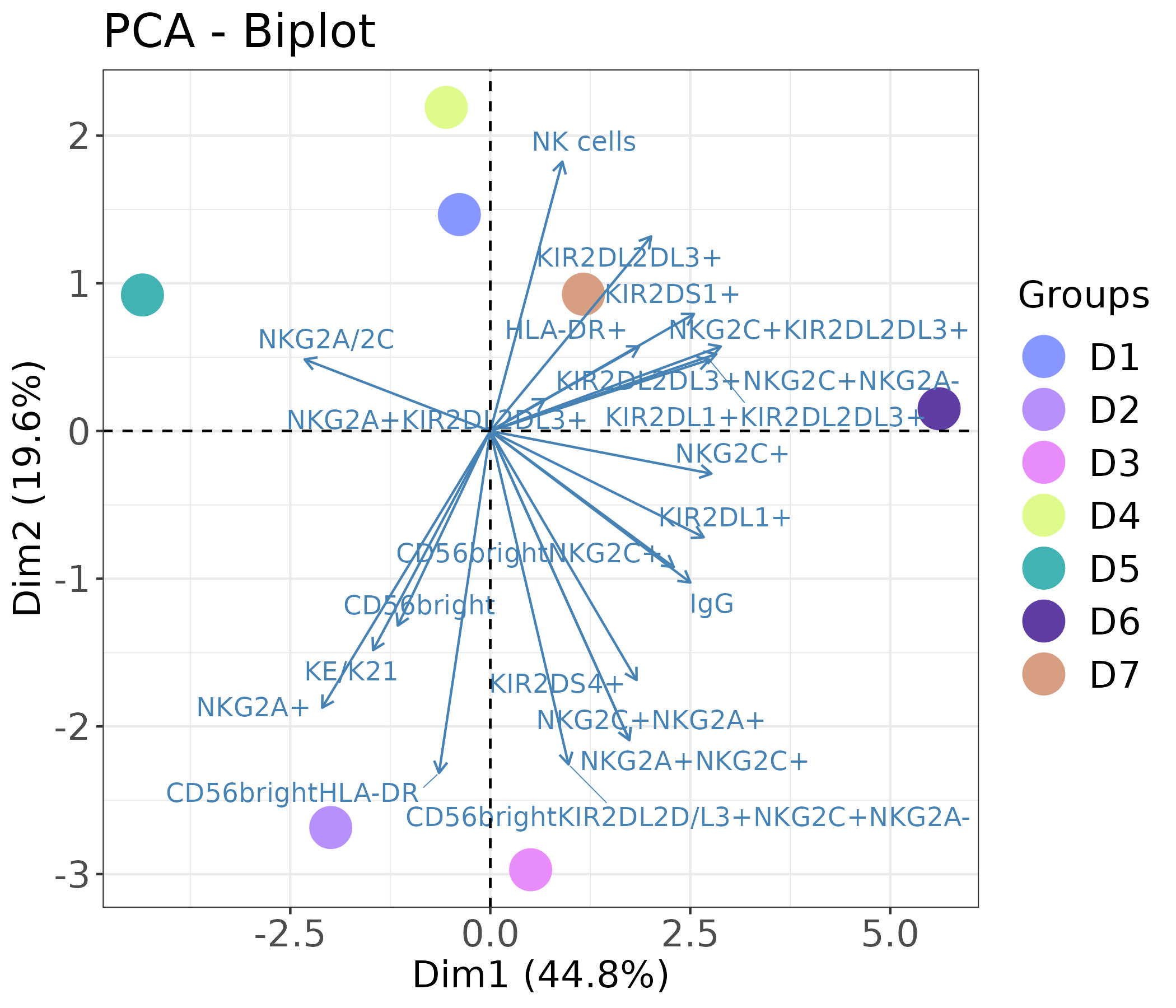

Supplement: Supplementary file 1 [file pharmaceutics-16-00133-s001.zip › Supplementary figures/Figure S3.jpg]

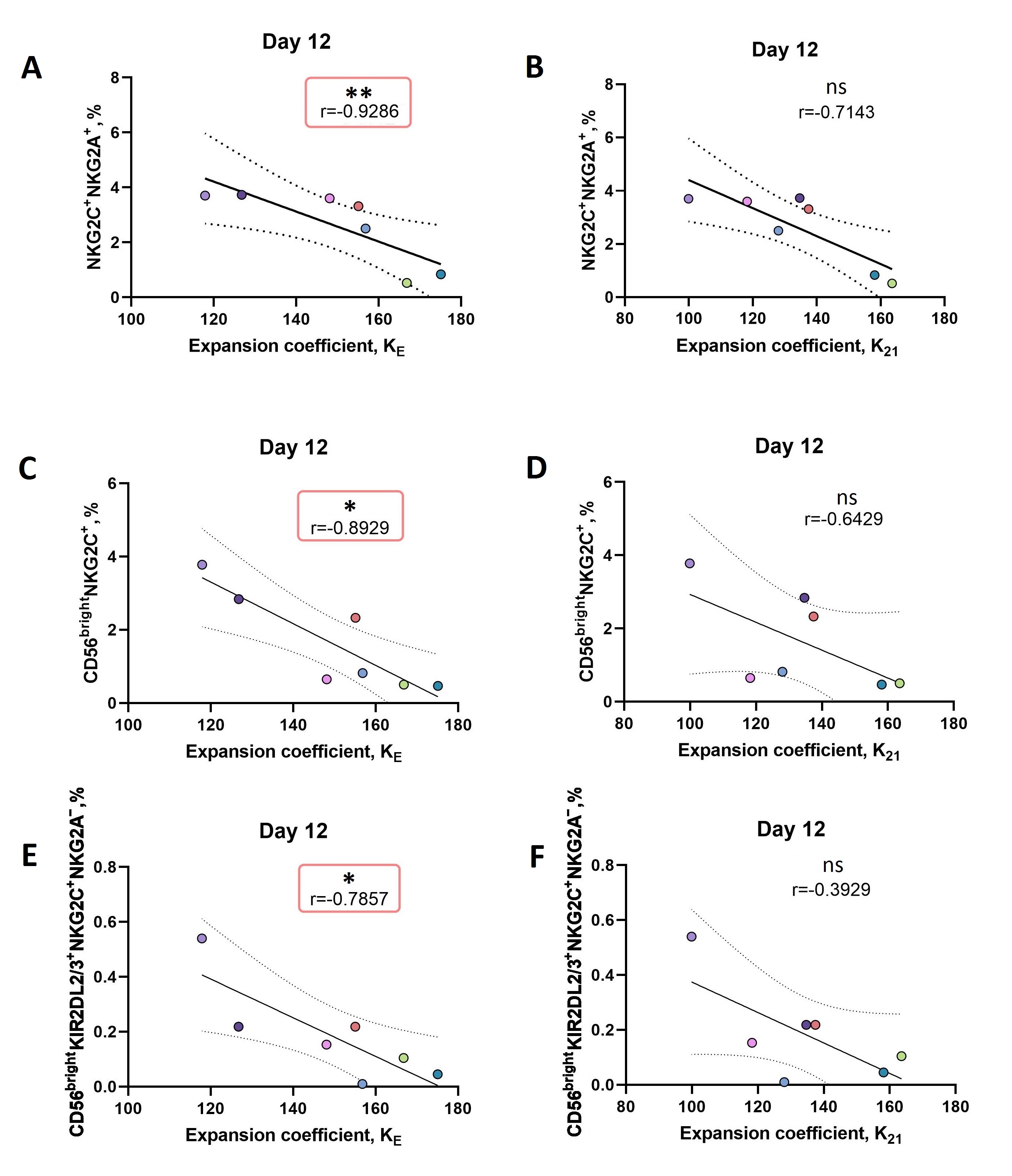

Supplement: Supplementary file 1 [file pharmaceutics-16-00133-s001.zip › Supplementary figures/Figure S4.jpg]

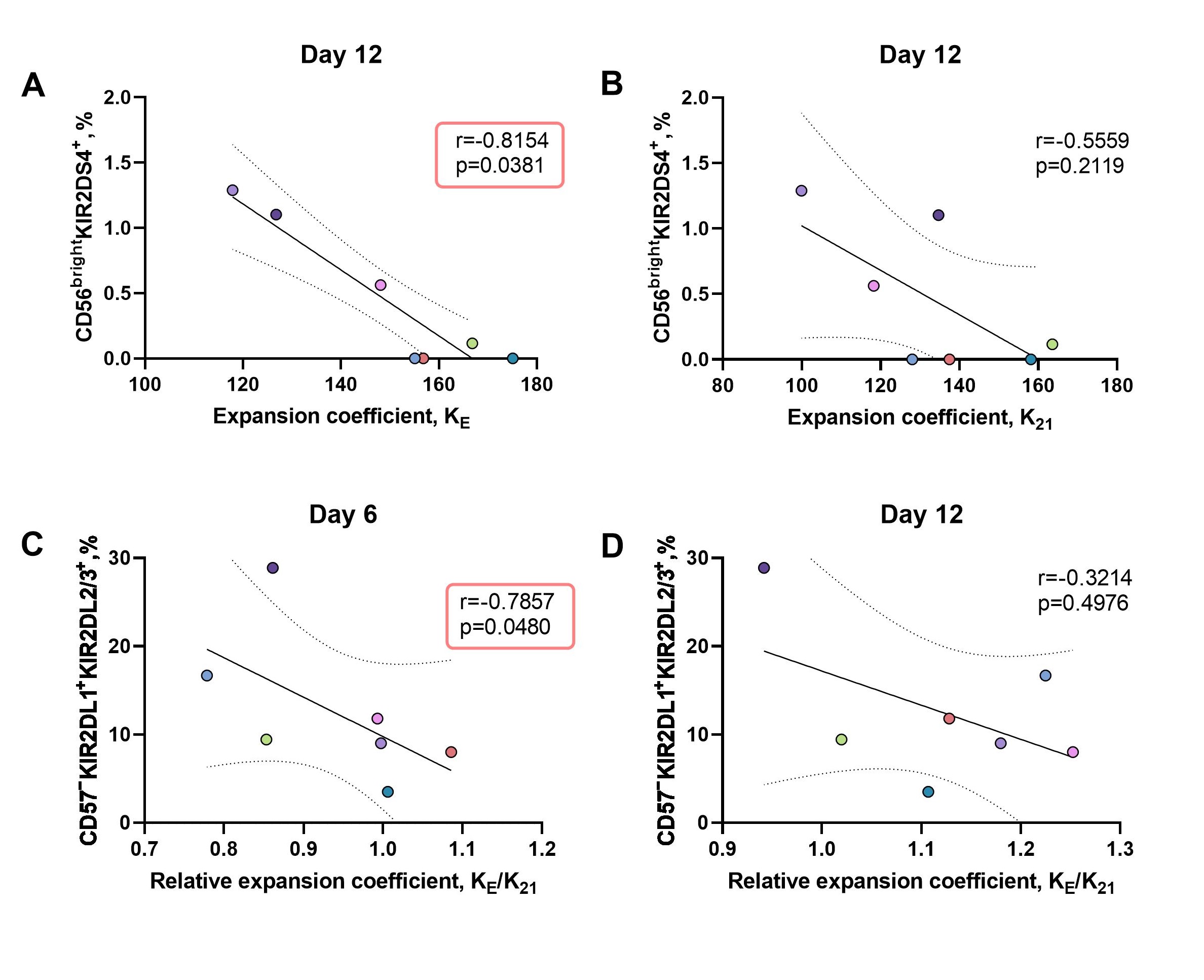

Supplement: Supplementary file 1 [file pharmaceutics-16-00133-s001.zip › Supplementary figures/Figure S5.jpg]

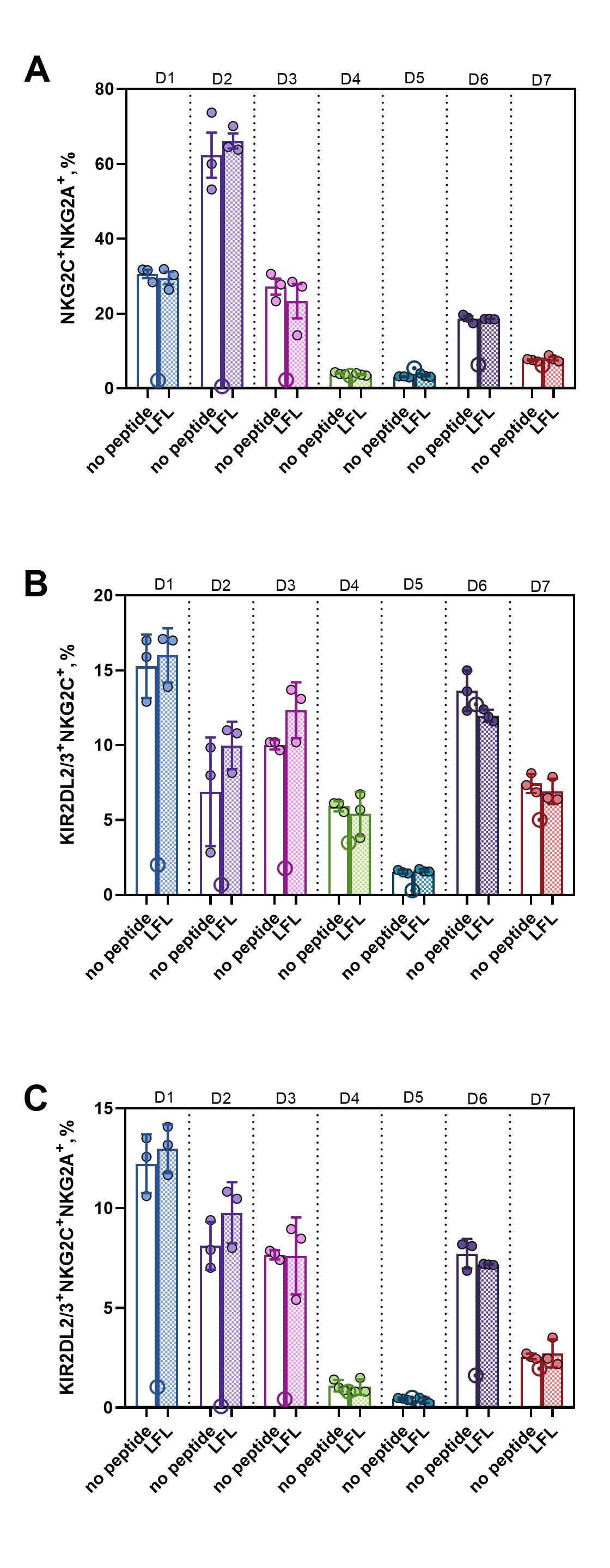

Supplement: Supplementary file 1 [file pharmaceutics-16-00133-s001.zip › Supplementary figures/Figure S6.jpg]

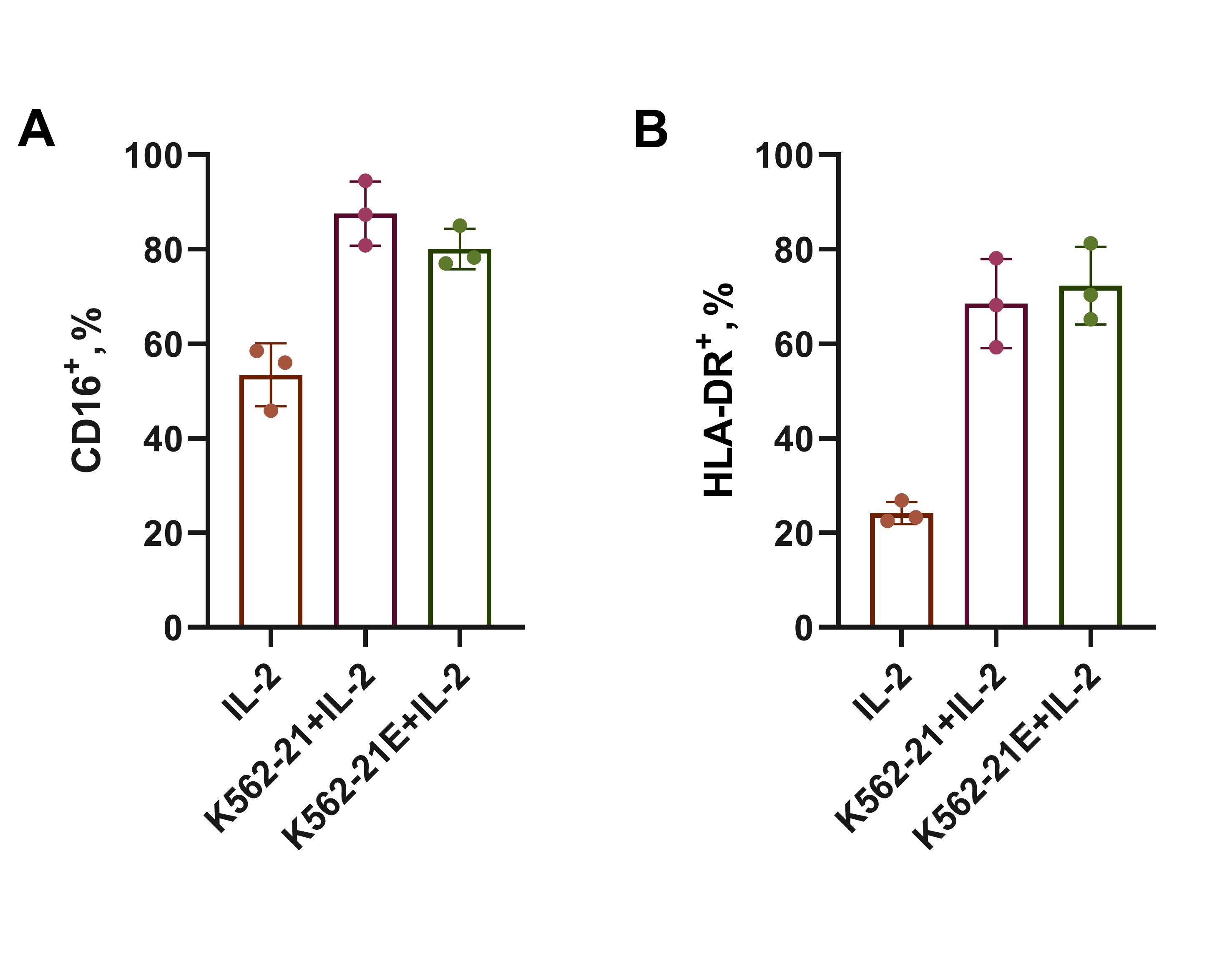

Supplement: Supplementary file 1 [file pharmaceutics-16-00133-s001.zip › Supplementary figures/Figure S1.jpg]

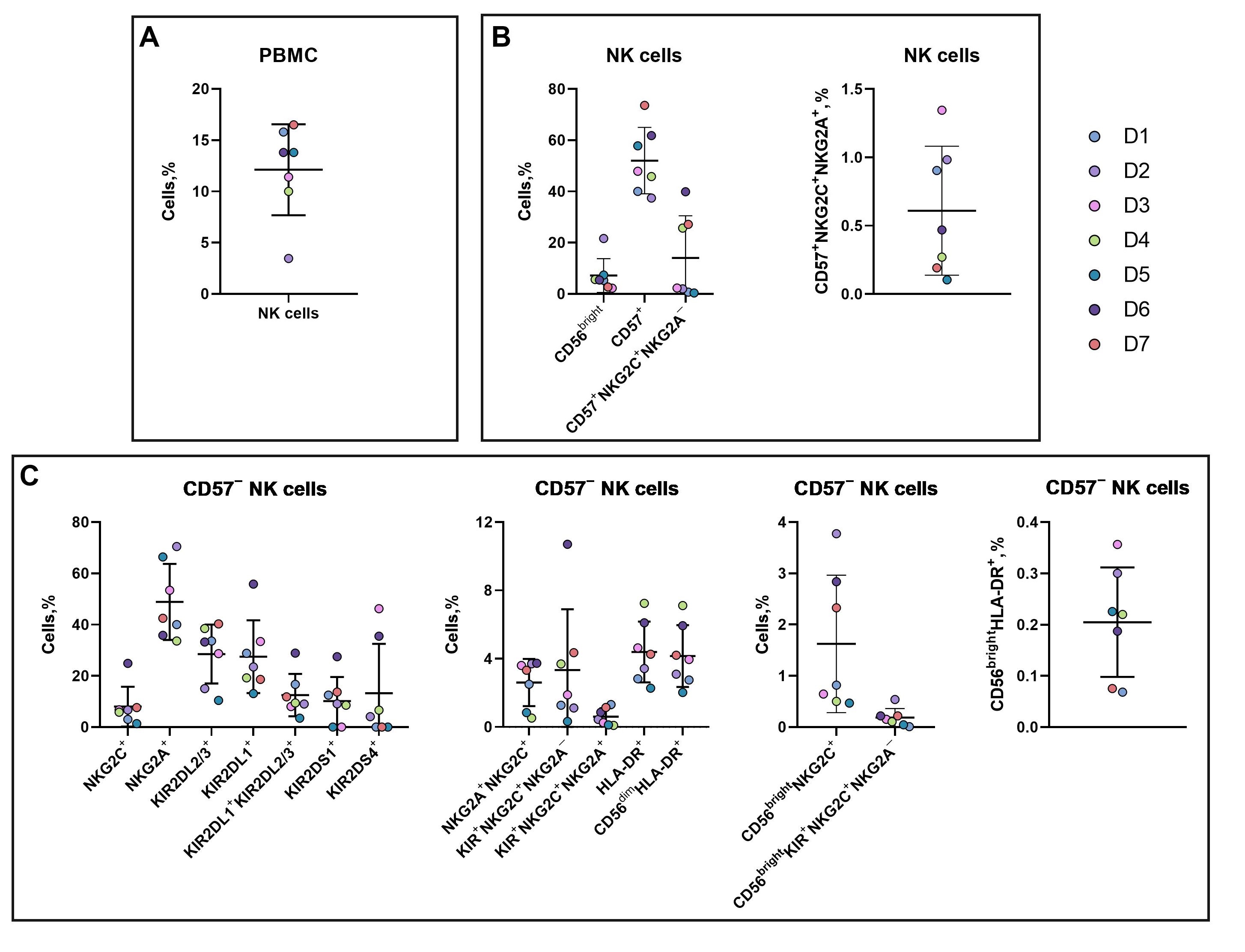

Supplement: Supplementary file 1 [file pharmaceutics-16-00133-s001.zip › Supplementary figures/Figure S2.jpg]
